# Gender-specific associations of speech-frequency hearing loss, high-frequency hearing loss, and cognitive impairment among older community dwellers in China

**DOI:** 10.1007/s40520-021-01990-0

**Published:** 2021-10-18

**Authors:** Jingru Wang, Feng Wang, Peipei Han, Yuewen Liu, Weibo Ma, Hui Zhang, Xing Yu, Fandi Xie, Shumeng Niu, Hao Hu, Xiaohan Zhu, Hongbing Wang, Ying Yu, Qi Guo

**Affiliations:** 1 Department of Rehabilitation Medicine, Shanghai University of Medicine and Health Sciences Affiliated Zhoupu Hospital, 1500 Zhouyuan Road, Shanghai, 201318 Pudong New District China; 2 Shanghai University of Medicine and Health Sciences, 279 Zhouzhu Highway, Shanghai, 201318 Pudong New District China; 3 Graduate School, Shanghai University of Traditional Chinese Medicine, Shanghai, China; 4 Shanghai Jiangwan Hospital, Shanghai, China; 5 Department of Rehabilitation, Shanghai Fourth Rehabilitation Hospital, Shanghai, China

**Keywords:** Hearing loss, High-frequency hearing loss, Speech-frequency hearing loss, Cognitive impairment, Gender difference

## Abstract

**Background and Aims:**

This study aimed atinvestigating the relationship between speech-frequency hearing loss (SFHL), high-frequency hearing loss (HFHL), and cognitive impairment (CI) and then to determine whether there are any differences in gender among older community dwellers in China.

**Methods:**

1012 adults aged ≥ 60 years (428 males; average age, 72.61 ± 5.51 years) and living in Chongming District, Shanghai were enrolled in the study. We used the audiometric definition of hearing loss (HL) adopted by the World Health Organization (WHO). Speech-frequencies were measured at 0.5 kHz, 1 kHz, 2 kHz, and 4 kHz; high-frequencies were measured at 4 kHz and 8 kHz. Pure tone average (PTA) was measured as hearing sensitivity. Cognitive performance was measured using the mini mental state examination (MMSE).

**Results:**

Our studies demonstrated a 37.6% prevalence of HL in males and a 36.0% prevalence of HL in females. Adjusted for confounding variables, the results from a multivariate analysis showed that SFHL was associated with CI in females (OR = 2.922, 95% Confidence Interval = 1.666–5.124) and males (OR = 2.559, 95% Confidence Interval = 1.252–5.232). However, HFHL was associated with CI only in females (OR = 3.490, 95% Confidence Interval = 1.834–6.643). HL was associated with poorer cognitive scores (P < 0.05). “Registration” (*P* < 0.05) in MMSE was associated with speech- and high-frequency hearing sensitivity.

**Conclusions:**

The associations between HL and CI varied according to gender in older community-dwellers, suggesting that different mechanisms are involved in the etiology of HL. Moreover, hearing sensitivity was negatively associated with cognition scores; therefore, early screening for HL and CI among older community-dwelling adults is advised.

## Introduction

Cognitive impairment (CI) is prevalent among older people, with nearly 43.8 million people having CI worldwide [1]. In China, the incidence of CI is 62.7% in females and 45.4% in males over 75 years of age, indicating a statistically significant gender difference [2]. The global costs for dementia are estimated to be $9.12 trillion (USD) in 2050 [3]. Dementia is a severe stage of CI with no modified treatments, so a focus on reducing modifiable risk factors is justified [4]. Many modifiable risk factors for CI have been identified, including social interactions, physical activity (PA), and years of formal education [5]. Evidence also indicates that hearing loss (HL) could be a risk factor [6, 7].

In recent years, more studies have concentrated on the relationship between HL and cognitive decline. Some researchers have proposed a strong correlation between HL and CI in older adults [8, 9]; however, others have not shown any association [10, 11]. The heterogeneity may be related to differences in cognitive tests and HL assessment methods. Presently, there is inconclusive evidence about their relationship, and the detailed mechanisms remain unknown. However, HL is reportedly associated with increased cognitive demand during speech perception [12]. HL most commonly encountered in older adults is age-related hearing loss (ARHL), which begins with high-frequency hearing loss (HFHL) and gradually affecting mid- and low-frequencies [13]. The clinical manifestation is speech perception difficulty in a noisy environment, which develops gradually to speech perception difficulty in a quiet environment. Speech perception is a process in which people hear, interpret and understand the sounds of language. Cognitive functions are instinctively involved in speech perception. Two cognitive factors decline with age, which may affect speech perception performance, namely working memory capacity and the rate of information processing [14]. Different-frequencies HL may be associated with CI, but few studies have investigated their link. Exploring the relationship between different frequencies HL and CI is helpful to reveal the influence of ARHL on CI in different stages of the disease and work toward a more targeted early intervention. Moreover, a few studies have revealed gender differences in HL [15, 16]; some studies have found that HL is more prevalent in males than females and that the decline in hearing thresholds at 6 kHz to 12 kHz was significantly rapid in females than males [17]. Aging also plays a role; for example, older adult females (> 70 years) demonstrated a faster rate of change at 0.25 kHz to 2 kHz than younger females (60–69 years), and older adult males had a faster rate of change at 6 kHz than younger males [17]. Since there is a protective role of estrogen with regard to HL, HL may play different roles on cognitive health by gender [16]. However, currently there is a paucity of research examining gender-specific associations between HL and CI in old age.

The purpose of this study was to analyze the associations between HL at different frequencies and CI in community-dwelling adults in China, in order to allow early screening for HL and CI to play a warning role and investigate whether there is a gender difference between HL and CI. Because HL and CI are two major geriatric health issues and are related to the quality of late life, determining gender differences in these associations will be helpful in developing gender-specific health policies that can contribute to the well-being of older adults.

## Materials and methods

### Study participants

This cross-sectional study was conducted in four different communities in Chongming District, Shanghai. We collected the physical examination data of older adults living in four different communities from June to July 2019, including demographic and health-related parameters (Table 1). We recruited a total of 1136 subjects aged 60 years and older. Finally, our study samples included 1012 subjects after excluding participants who (a) had mental illness or other neurodegenerative diseases; (b) were diagnosed with dementia; (c) had hearing aids; (d) had a medical history of sudden deafness, otitis media, otitis externa, ototoxic drug therapy, and otologic surgery; (e) had missing data; (f) had extreme value in physical performance; and (g) were unable to communicate with interviewers or grant informed consent.

**Table 1:** The characters of the participants. (N = 1012)

Variables	ALL			Male			Female		
	Normal	HL	*P*	Normal	HL	*P*	Normal	HL	*P*
	(*N* = 641)	(*N* = 371)		(*N* = 267)	(*N* = 161)		(*N* = 374)	(*N* = 210)	
Age (y)	70.99 ± 4.71	75.38 ± 6.25	**0.000**	71.42 ± 4.77	74.57 ± 6.08	**0.000**	70.68 ± 4.65	76.00 ± 6.32	**0.000**
Male (%)	267 (41.65)	161 (43.40)	0.589	–	–	–	–	–	–
Speech-PTA (dB HL)	37.14 ± 7.43	56.75 ± 9.05	**0.000**	37.03 ± 7.10	56.54 ± 8.66	**0.000**	37.21 ± 7.67	56.92 ± 9.36	**0.000**
High-PTA (dB HL)	37.23 ± 7.70	60.72 ± 9.86	**0.000**	37.50 ± 7.48	61.52 ± 9.69	**0.000**	37.07 ± 7.83	60.02 ± 9.94	**0.000**
BMI (kg/m^2^)	23.77 ± 3.38	23.50 ± 3.76	0.246	23.40 ± 3.28	23.14 ± 3.79	0.457	24.04 ± 3.44	23.77 ± 3.72	0.379
SBP (mmHg)	129.66 ± 18.90	133.00 ± 21.16	**0.012**	127.54 ± 17.88	130.97 ± 19.47	0.065	131.19 ± 19.48	134.57 ± 22.29	0.068
DBP (mmHg)	73.17 ± 10.45	73.40 ± 11.54	0.741	74.26 ± 10.26	75.19 ± 10.81	0.373	72.38 ± 10.53	72.02 ± 11.92	0.709
Marital status (%)			**0.000**			0.533			**0.000**
Married	529 (82.53)	254 (68.4)		243 (91.01)	141 (87.58)		286 (76.47)	113 (53.81)	
Windowed	109 (17.00)	115 (30.99)		22 (8.24)	19 (11.8)		87 (23.26)	96 (45.71)	
Single	1 (0.16)	2 (0.54)		1 (0.37)	1 (0.62)		0 (0.00)	1 (0.48)	
Divorced	2 (0.31)	0 (0.00)		1 (0.37)	0 (0.00)		1 (0.27)	0 (0.00)	
Living alone (%)	92 (14.40)	86 (23.20)	**0.000**	28 (10.50)	24 (14.90)	0.175	64 (17.10)	62 (29.50)	**0.000**
Monthly income (%)			**0.030**			0.081			0.226
< 1000	54 (8.40)	44 (11.90)		17 (6.39)	13 (8.13)		37 (9.89)	31 (14.83)	
1000–3000	367 (57.30)	229 (62.10)		134 (50.38)	98 (61.25)		233 (62.3)	131 (62.68)	
3000–5000	97 (15.20)	44 (11.90)		51 (19.17)	22 (13.75)		46 (12.30)	22 (10.53)	
> 5000	122 (19.10)	52 (14.10)		64 (24.00)	27 (16.88)		58 (15.51)	25 (11.96)	
Education (%)			**0.000**			0.411			**0.000**
Illiteracy	70 (10.92)	84 (22.64)		15 (5.62)	1 0(6.21)		55 (14.71)	74 (35.24)	
Primary	375 (58.50)	200 (53.91)		135 (50.56)	91 (56.52)		240 (64.17)	109 (51.90)	
≥ High school	196 (30.58)	87 (23.45)		117 (43.82)	60 (37.27)		79 (21.12)	27 (12.86)	
Smoking (%)			0.682			0.650			0.288
Current	99 (15.47)	54 (14.59)		98 (36.70)	52 (32.30)		1 (0.27)	2(0.96)	
Never	440 (68.75)	250 (67.57)		69 (25.84)	45 (27.95)		371 (99.46)	205 (98.09)	
Former	101 (15.78)	66 (17.84)		100 (37.45)	64 (39.75)		1 (0.27)	2 (0.96)	
Drinking (%)			0.086			0.402			**0.010**
Daily	88 (13.77)	55 (14.86)		75 (28.2)	37(23.13)		13 (3.49)	18 (8.57)	
Occasional	95 (14.87)	52 (14.05)		56 (21.05)	32 (20.00)		39 (10.46)	20 (9.52)	
Former	67 (10.49)	58 (15.67)		54 (20.30)	43 (26.88)		13 (3.49)	15 (7.14)	
Never	389 (60.88)	205 (55.41)		81 (30.45)	48 (30.00)		308 (82.57)	157 (74.76)	
Disease history (%)									
Diabetes	130 (21.74)	77 (22.92)	0.678	48 (19.12)	41 (27.52)	0.051	82 (23.63)	36 (19.25)	0.245
Hypertension	457 (71.20)	279 (75.41)	0.157	191 (71.54)	111 (69.38)	0.635	266 (71.12)	168 (80.00)	**0.018**
Hyperlipidemia	305 (54.17)	167 (52.68)	0.670	97 (42.17)	58 (41.43)	0.888	208 (62.46)	109 (61.58)	0.845
Stroke	32 (5.09)	32 (8.79)	**0.022**	23 (8.75)	10 (6.29)	0.363	9 (2.46)	22 (10.73)	**0.000**
MMSE	25.73 ± 4.04	22.77 ± 5.55	**0.000**	26.54 ± 3.67	24.58 ± 4.03	**0.000**	25.16 ± 4.19	21.38 ± 6.13	**0.000**
IPAQ (Met/week)	4746 (1680,9786)	4620 (1606,9786)	0.985	4053 (1440,8022)	5040 (1533,10,733)	0.061	5239 (2140,10,600)	415 5(1621,9056)	**0.023**
ADL	99.49 ± 2.19	98.91 ± 3.76	**0.006**	99.64 ± 1.49	98.79 ± 4.46	**0.020**	99.38 ± 2.57	99 ± 3.12	0.131
IADL	7.71 ± 0.74	7.34 ± 1.34	**0.000**	7.69 ± 0.76	7.51 ± 1.15	0.085	7.73 ± 0.72	7.21 ± 1.46	**0.000**

Note: Significant probabilities are marked in bold

Abbreviation: *PTA* pure-tone average, *High PTA* was defined as pure tone average of the threshold at 4, and 8 kHz in the better ear, *Speech PTA* was defined as pure tone average of the threshold at 0.5, 1, 2, and 4 kHz in the better ear, *HL* hearing loss, and was defined as PTA_(0.5,1,2, and 4 kHz)_ more than 40 dB loss in the better ear, *BMI* body mass index, *SBP* systolic blood pressure, *DBP* diastolic pressure, *IPAQ* international physical activity questionnaire, *ADL* activity of daily living, *IADL* instrumental activity of daily living, *MMSE* mini-mental status examination

This study was conducted in accordance with the recommendations of national and international guidelines, and our ethical committee. All of the subjects gave their written informed consent in accordance with the Declaration of Helsinki. The protocol was approved by the Ethical Committee of the Shanghai University of Medicine and Health Sciences.

### Hearing assessment

The subjects’ hearing was measured using pure tone audiometry (BTJ09; JiangSu BetterLife Medical Co., Ltd, China.). Air conduction thresholds (dB) were measured for both ears at seven frequencies (0.125 kHz, 0.25 kHz, 0.5 kHz, 1 kHz, 2 kHz, 4 kHz, and 8 kHz), and across an intensity range of 0 dB to 100 dB, but we did not measure 6 kHz for the limited time. The speech-frequency pure tone average (PTA) was computed as mean thresholds at 0.5 kHz, 1 kHz, 2 kHz, and 4 kHz, which are most important and represent speech perception [18]. The high-frequency PTA was computed as mean thresholds at 4 kHz and 8 kHz. Pure tone threshold averages in the better ear were calculated to identify grades of hearing disorder in adults according to the World Health Organization (WHO) Prevention of Deafness and Hearing Impairment (PDH) standard 97.3 [19]. The speech-frequency hearing loss (SFHL) represents hearing loss from the WHO [19]. Defined as more than 40 dB loss in the better ear. The HFHL was also defined as more than 40 dB loss in the better ear.

### Cognition assessment

Cognitive performance was measured using the mini mental state examination (MMSE). The MMSE items assess several cognitive domains, which are summed to a maximum total score of 30 points. The items may be clustered in six domains measuring different cognitive processes: orientation to time (5 points), orientation to place (5 points), registration (3 points), attention (5 points), recall (3 points), and language (9 points). Considering the participants’ education, CI was defined as MMSE ≤ 17 for illiterates; MMSE ≤ 20 for primary school graduates; and MMSE ≤ 24 for junior high school graduates or those with higher-level education [20]. It is worth noting that a correlation between better cognitive performance (higher MMSE score) and better hearing (lower thresholds) is represented by a negative correlation coefficient.

### Other covariates

All of the participants were asked to complete a questionnaire by face-to-face interviews, physical examination, and blood sample collection during baseline. The data on sociodemographic characteristics, behavioral characteristics, and medical history were obtained with questionnaires. The sociodemographic characteristics included sex, age, education level, monthly income, living status, and marital status. Height and weight were measured, and body mass index (BMI) was calculated as weight in kilograms divided by the square of height in meters. Education level was categorized as illiterates, primary school graduation, high school graduation and above. Monthly income was categorized as < 1000 yuan, 1000–3000 yuan, 3000–5000 yuan, and > 5000 yuan. Living alone was categorized as yes or no. Marital status was categorized as married, widowed, single, and divorced. The behavioral characteristics included smoking history, drinking history, PA situation, Activity of daily living scale (ADL), and Instrumental activity of daily living (IADL). Smoking history was categorized as follows: nonsmokers, former smokers, and current smokers. Drinking history was also categorized as follows: nondrinkers, former drinkers, and current drinkers. PA and sitting time in the most recent week were assessed using the short form of the International Physical Activity Questionnaire (IPAQ) [21]. Activity of daily living was assessed as ADL [22] and IADL [23]. Medical history included hypertension, diabetes, hyperlipidemia, and stroke. Hypertension was defined as systolic blood pressure ≥ 140 mmHg or diastolic blood pressure ≥ 90 mmHg or self-reported diagnosis and current use of antihypertensive medication [24]. Diabetes was defined as fasting plasma glucose of ≥ 7.0 mmol/L or self-reported diagnosis and current use of insulin or other medications for diabetes [25]. Hyperlipidemia was defined as self-reported diagnosis and current use of antihyperlipidemic medication. Stroke was defined as self-reported history.

### Statistical analysis

All statistical analyses were performed using the SPSS 25.0 edition for Microsoft Windows (SPSS Institute Inc., Chicago, IL, USA). The descriptive characteristics for categorical variables were summarized as percentages, and significant differences were evaluated using a χ^2^ test. Continuous variables were summarized as mean ± SD or median (interquartile range) values, and comparisons were performed using the *t*-test. A multiple logistic regression model was used to examine the association of different frequency hearing loss with CI. The model was stratified according to gender and age, respectively. We generated a model adjusted for BMI, marital status, living alone, monthly income, education, IPAQ, ADL, IADL, diabetes, hypertension, hyperlipidemia, and stroke. The linear regression models were adjusted for age and above; the association of different frequencies of hearing sensitivity with cognitive domains (time, place, registration, recall, attention and calculation, and language) was evaluated. All of the tests were two-tailed, and the differences were considered to be statistically significant at *P* < 0.05.

## Results

### Study sample

**Fig. 1:**
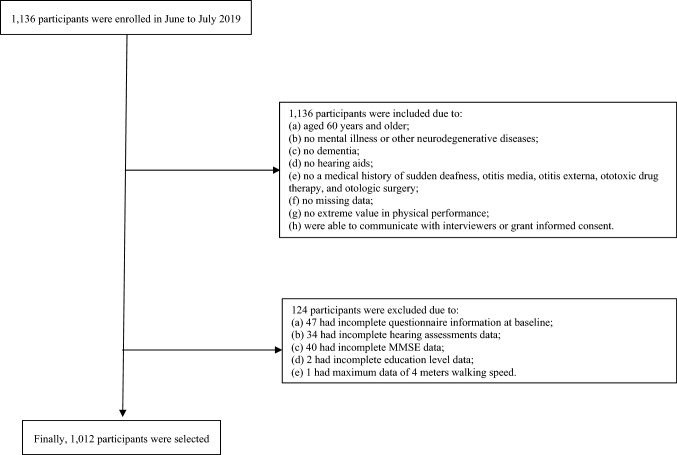
Study flow diagram

A total of 1,136 participants were evaluated in person by study personnel. Of these, 124 were excluded: 47 had incomplete questionnaire information at baseline, 34 had incomplete hearing assessments data, 40 had incomplete MMSE data, 2 had incomplete education level data, and 1 had maximum data of 4 m walking speed (Fig. 1).

### Participant characteristics

The demographic characteristics and the distribution of different covariates between the normal hearing and HL groups are described in Table 1. Of 1012 people included in the analyses, 371 had HL (43.4% males) and 641 (41.7% males) had normal hearing. The PTA in the better ear for speech-frequencies 0.5–4 kHz was 56.54 dB HL (± 8.66) in males with HL and 56.92 dB HL (± 9.36) in females with HL; the high-frequency 4–8 kHz PTA was 61.52 dB HL (± 9.69) in males with HL and 60.02 dB HL (± 9.94) in females with HL. Compared to males with normal hearing, males with HL were more likely to be older (71.42 years vs 74.57 years; *P* < 0.001), and had lower MMSE scores (26.54 vs 24.58; *P* < 0.001). These characteristics were also evident in females. Moreover, there are different associations by gender. For example, the HL in females showed a relationship with marital status (*P* < 0.001), living alone (17.1% vs 29.5%; *P* < 0.001), education level (*P* < 0.001), and drinking history (*P* = 0.010), but these results are not significant in males (all *P* > 0.05). Meanwhile, compared to those with normal hearing, those with HL did not demonstrate differences in terms of smoking history among males and females (*P* > 0.05).

### Association of CI with HL

Table 2 presents the association of HL with CI. Compared to males with normal hearing, the SFHL in males was associated with CI in model 1 with unadjusted covariates (odds ratio [OR]: 2.600, 95% Confidence Interval: 1.411–4.791). After adjusting the model for covariates (BMI, marital status, living alone, monthly income, education, IPAQ, ADL, IADL, diabetes, hypertension, hyperlipidemia, and stroke), we still found a statistically significant association between them (OR: 2.559, 95% Confidence Interval: 1.252–5.232). Moreover, the statistically significant association between HFHL and CI disappeared in all of models. In females, CI was associated with both SFHL and HFHL in model 1 with unadjusted covariates (OR: 4.872, 95% Confidence Interval: 3.154–7.526; OR: 6.093, 95% Confidence Interval: 3.530–10.518, respectively). We also found a statistically significant association between them (OR: 2.922, 95% Confidence Interval: 1.666–5.124; OR: 3.490, 95% Confidence Interval: 1.834–6.643, respectively), even after adjusted for many covariates (BMI, marital status, living alone, monthly income, education, IPAQ, ADL, IADL, diabetes, hypertension, hyperlipidemia, and stroke).

**Table 2:** Logistic regression between hearing loss and cognitive impairment, stratified by gender

Variables	Model 1		Model 2	
	OR (95%CI)	*P*	OR (95%CI)	*P*
ALL				
HFHL	3.851 (2.508,5.914)	**0.000**	2.548 (1.565–4.148)	**0.000**
SFHL	3.842 (2.706,5.457)	**0.000**	2.763 (1.814–4.208)	**0.000**
Male				
HFHL	1.875 (0.926,3.794)	0.081	1.488 (0.683–3.243)	0.317
SFHL	2.600 (1.411,4.791)	**0.002**	2.559 (1.252–5.232)	**0.010**
Female				
HFHL	6.093 (3.53,10.518)	**0.000**	3.490 (1.834–6.643)	**0.000**
SFHL	4.872 (3.154,7.526)	**0.000**	2.922 (1.666–5.124)	**0.000**

Note: Significant probabilities are marked in bold

*HFHL* high-frequency hearing loss, *HFHL* was defined as more than 40 dB loss in the better ear

*SFHL* speech-frequency hearing loss, *SFHL* was defined as more than 40 dB loss in the better ear

Model 1: unadjusted model

Model 2: adjusted for BMI, marital status, living alone, monthly income, education, drinking, smoking, IPAQ, ADL, IADL, diabetes, hypertension, hyperlipidemia, and stroke

In order to ensure the reliability of our research results, we conducted a stratified analysis by age to assess possible effect modification on the association between SFHL, HFHL and CI. Table 3 presents the 2 models for the association between SFHL, HFHL and CI in males and in females. Model 2 in females showed that SFHL were significantly associated with CI between ages 65 and 74 (all *P* < 0.05), and HFHL were significantly associated with CI between ages 65 and 69 (*P* = 0.009); no similar results were observed in the 75 years and older group. However, in males, none of the models showed significant associations of HFHL or SFHL with CI in all age groups.

**Table 3:** Logistic regression between hearing loss and cognitive impairment, stratified by age

Variables	65–69 years		70–74 years		≥ 75 years	
	OR (95%CI)	*P*	OR (95%CI)	*P*	OR (95%CI)	*P*
*Male (n)*	144		143		135	
*SFHL*						
Model 1	3.323 (0.903–12.228)	0.071	1.412 (0.424–4.706)	0.574	2.05 3(0.823–5.118)	0.123
Model 2	1.319 (0.101–17.162)	0.832	0.905 (0.133–6.158)	0.919	2.626 (0.781–8.824)	0.119
*HFHL*						
Model 1	1.331 (0.359–4.932)	0.669	2.529 (0.531–12.044)	0.244	1.316 (0.451–3.836)	0.615
Model 2	0.564 (0.037–8.527)	0.680	1.943 (0.284–13.310)	0.499	1.345 (0.328–5.512)	0.681
*Female (n)*	201		167		191	
*SFHL*						
Model 1	8.050 (2.387–27.152)	**0.001**	5.643 (2.034–15.654)	**0.001**	2.758 (1.449–5.248)	**0.002**
Model 2	9.739 (1.922–49.360)	**0.006**	5.827 (1.247–27.233)	**0.025**	2.062 (0.911–4.663)	0.082
*HFHL*						
Model 1	10.242 (2.178–48.170)	**0.003**	3.636 (1.160–11.398)	**0.027**	3.875 (1.528–9.827)	**0.004**
Model 2	9.832 (1.766–54.737)	**0.009**	2.783 (0.580–13.355)	0.201	2.797 (0.907–8.623)	0.073

Note: Significant probabilities are marked in bold

*PTA* pure-tone average, High PTA was defined as pure tone average of the threshold at 4, and 8 kHz in the better ear; Speech PTA was defined as pure tone average of the threshold at 0.5, 1, 2, and 4 kHz in the better ear

Model 1: unadjusted model

Model 2: adjusted for BMI, marital status, living alone, monthly income, education, drinking, smoking, IPAQ, ADL, IADL, diabetes, hypertension, hyperlipidemia, and stroke

### Association of cognition with hearing sensitivity

To determine the independent associations between cognition and hearing sensitivity of different frequencies, Tables 4, 5, and 6, respectively, showed linear regression between PTA and MMSE domains of CI by gender. We found in these tables that hearing sensitivity negatively affected the cognitive function (all *P* < 0.05) in model 1. In addition, there was a significant negative correlation between hearing sensitivity and cognitive domains (time, place, registration, recall, attention/calculation, and language) (all *P* < 0.05). After adjusting for covariates (age, BMI, marital status, living alone, monthly income, education, IPAQ, ADL, IADL, diabetes, hypertension, hyperlipidemia, and stroke), the association had changed in some cognitive domains. Table 4 shows that speech-frequencies PTA negatively affected all cognitive domains (all *P* < 0.05) in model 2. High-frequency PTA negatively affected all cognitive domains in model 2, except the orientation of place (*P* = 0.099) and time (*P* = 0.065). In addition, Table 5 shows a linear regression between hearing sensitivity and cognitive domains in males. We found that high-frequency PTA affected negatively only the registration of five cognitive domains (*P* = 0.001) in model 2, while speech-frequency PTA affected the cognitive domains in model 2, except the orientation of place (*P* = 0.597) and language function (*P* = 0.072). Table 6 shows a linear regression between hearing sensitivity and cognitive domains in females. We found that high-frequency PTA negatively affected the orientation of time and language (all *P* < 0.05) in the unadjusted model, but after adjusting for confounding variates, these correlations disappeared. Moreover, there was an association in speech-frequencies PTA involving the orientation of place (*P* = 0.003), attention/calculation (*P* = 0.003), and registration (P = 0.003). Obviously, registration was associated with high- and speech-frequency PTA in all models. It is worth noting that this also existed in males.

**Table 4:** Linear regression between hearing sensitivity and cognitive impairment

Variables	Model 1		Model 2	
	β (95%CI)	*P*	β (95%CI)	*P*
*High PTA*				
MMSE	– 0.084 ( – 0.102, – 0.066)	**0.000**	– 0.038 ( – 0.055, – 0.022)	**0.000**
Time	– 0.014 ( – 0.018, – 0.010)	**0.000**	– 0.004 ( – 0.009, 0.000)	0.065
Place	– 0.010 ( – 0.013, – 0.006)	**0.000**	– 0.003 ( – 0.007, 0.001)	0.099
Registration	– 0.008 ( – 0.011, – 0.006)	**0.000**	– 0.007 ( – 0.009, – 0.004)	**0.000**
Recall	– 0.013 ( – 0.018, – 0.009)	**0.000**	– 0.008 ( – 0.014, – 0.003)	**0.002**
Attention/calculation	– 0.021 ( – 0.027, – 0.014)	**0.000**	– 0.012 ( – 0.019, – 0.003)	**0.001**
Language	– 0.019 ( – 0.023, – 0.014)	**0.000**	– 0.004 ( – 0.009, 0.000)	**0.050**
*Speech PTA*				
MMSE	– 0.131 ( – 0.151, – 0.111)	**0.000**	– 0.061 ( – 0.081, – 0.042)	**0.000**
Time	– 0.021 ( – 0.026, – 0.016)	**0.000**	– 0.008 (-0.014, – 0.003)	**0.003**
Place	– 0.017 ( – 0.021, – 0.013)	**0.000**	– 0.007 ( – 0.011, – 0.002)	**0.003**
Registration	– 0.012 ( – 0.014, – 0.009)	**0.000**	– 0.009 ( – 0.012, – 0.005)	**0.000**
Recall	– 0.019 ( – 0.025, – 0.014)	**0.000**	– 0.011 ( – 0.017, – 0.004)	**0.001**
Attention/calculation	– 0.033 ( – 0.040, – 0.026)	**0.000**	– 0.020 ( – 0.028, – 0.012)	**0.000**
Language	– 0.029 ( – 0.035, – 0.023)	**0.000**	– 0.007 ( – 0.013, – 0.002)	**0.010**

Note: Significant probabilities are marked in bold

*PTA* pure-tone average, High PTA was defined as pure tone average of the threshold at 4, and 8 kHz in the better ear; Speech PTA was defined as pure tone average of the threshold at 0.5, 1, 2, and 4 kHz in the better ear

Model 1: unadjusted

Model 2: adjusted for age, BMI, marital status, living alone, monthly income, education, drinking, smoking, IPAQ, ADL, IADL, diabetes, hypertension, hyperlipidemia, and stroke

**Table 5:** Linear regression between hearing sensitivity and cognitive impairment in males

Variables	Model 1		Model 2	
	β (95%CI)	*P*	β (95%CI)	*P*
*High PTA*				
MMSE	– 0.042 ( – 0.064, – 0.019)	**0.000**	– 0.027 ( – 0.051, 0.003)	**0.027**
Time	– 0.006 ( – 0.012, 0.000)	0.059	– 0.002 ( – 0.009, 0.005)	0.539
Place	– 0.002 ( – 0.005, 0.001)	0.242	0.000 ( – 0.003, 0.004)	0.835
Registration	– 0.008 (-0.011, – 0.004)	**0.000**	– 0.007 ( – 0.012, – 0.003)	**0.001**
Recall	– 0.008 ( – 0.015, – 0.000)	**0.037**	– 0.008 ( – 0.016, 0.000)	0.064
Attention/calculation	– 0.011( – 0.020, – 0.003)	**0.011**	– 0.009 ( – 0.018, 0.001)	0.078
Language	– 0.007 ( – 0.014, – 0.001)	**0.024**	– 0.002 ( – 0.008, 0.005)	0.577
*Speech PTA*				
MMSE	– 0.082 ( – 0.108, – 0.055)	**0.000**	– 0.066 ( – 0.095, – 0.037)	**0.000**
Time	– 0.012 ( – 0.019, – 0.005)	**0.001**	– 0.009 ( – 0.018, – 0.000)	**0.025**
Place	– 0.005 ( – 0.009, – 0.001)	**0.021**	– 0.001 ( – 0.006, 0.003)	0.597
Registration	– 0.012 ( – 0.016, – 0.007)	**0.000**	– 0.011 ( – 0.016, -0.006)	**0.000**
Recall	– 0.017 ( – 0.026, – 0.008)	**0.000**	– 0.015 ( – 0.025, – 0.005)	**0.003**
Attention/calculation	– 0.022 ( – 0.033, – 0.012)	**0.000**	– 0.022 ( – 0.033, – 0.010)	**0.000**
Language	– 0.014 ( – 0.021, – 0.006)	**0.000**	– 0.007 ( – 0.015, 0.001)	0.072

Note: Significant probabilities are marked in bold

*PTA* pure-tone average, *High PTA* was defined as pure tone average of the threshold at 4, and 8 kHz in the better ear; Speech PTA was defined as pure tone average of the threshold at 0.5, 1, 2, and 4 kHz in the better ear

Model 1: unadjusted

Model 2: adjusted for age, BMI, marital status, living alone, monthly income, education, drinking, smoking, IPAQ, ADL, IADL, diabetes, hypertension, hyperlipidemia, and stroke

**Table 6:** Linear regression between hearing sensitivity and cognitive impairment in females

Variables	Model 1		Model 2	
	β (95%CI)	*P*	β (95%CI)	*P*
*High PTA*				
MMSE	– 0.130 ( – 0.155, – 0.106)	**0.000**	– 0.047 ( – 0.071, – 0.024)	**0.000**
Time	– 0.022 ( – 0.027, – 0.016)	**0.000**	– 0.004 ( – 0.010, 0.003)	0.158
Place	– 0.019 ( – 0.024, – 0.014)	**0.000**	– 0.007 ( – 0.012, – 0.001)	**0.031**
Registration	– 0.009 ( – 0.013, – 0.006)	**0.000**	– 0.007 (-0.011, – 0.002)	**0.001**
Recall	– 0.018 ( – 0.023, – 0.012)	**0.000**	– 0.009 ( – 0.017, – 0.002)	**0.020**
Attention/calculation	– 0.032 ( – 0.041, – 0.023)	**0.000**	– 0.015 ( – 0.025, – 0.004)	**0.001**
Language	– 0.031 ( – 0.038, – 0.024)	**0.000**	– 0.006 ( – 0.013, 0.000)	0.062
*Speech PTA*				
MMSE	– 0.165 ( – 0.193, – 0.137)	**0.000**	– 0.055 ( – 0.082, – 0.027)	**0.000**
Time	– 0.027 ( – 0.033, – 0.020)	**0.000**	– 0.005 ( – 0.012, 0.003)	0.110
Place	– 0.026 ( – 0.032, – 0.020)	**0.000**	– 0.010 ( – 0.017, – 0.003)	**0.003**
Registration	– 0.012 ( – 0.015, – 0.008)	**0.000**	– 0.007 ( – 0.012, – 0.003)	**0.003**
Recall	– 0.021 ( – 0.028, – 0.014)	**0.000**	– 0.009 ( – 0.018, – 0.000)	0.053
Attention/calculation	– 0.040 ( – 0.050, – 0.030)	**0.000**	– 0.016 ( – 0.028, – 0.004)	**0.003**
Language	– 0.039 ( – 0.047, – 0.031)	**0.000**	– 0.007 ( – 0.014, 0.001)	0.117

Note: Significant probabilities are marked in bold

*PTA* pure-tone average, *High PTA* was defined as pure tone average of the threshold at 4, and 8 kHz in the better ear; Speech PTA was defined as pure tone average of the threshold at 0.5, 1, 2, and 4 kHz in the better ear

Model 1: unadjusted

Model 2: adjusted for age, BMI, marital status, living alone, monthly income, education, drinking, smoking, IPAQ, ADL, IADL, diabetes, hypertension, hyperlipidemia, and stroke

## Discussion

The purpose of our study was to investigate the relationship between SFHL, HFHL, and CI, then to determine whether any association differs by gender among older community dwellers in China. Our cross-sectional study results showed that SFHL and HFHL may be associated with a higher risk of CI. Furthermore, we found that SFHL was more strongly associated with CI in males and females, while HFHL had a different association with CI among males and females. Hearing sensitivity was negatively and independently related to MMSE scores, regardless of gender. We explored which cognitive domains on the MMSE are more strongly associated with hearing sensitivity stratified by gender. As far as we know, this is the first study to explore the different relationship in different-frequency HL and CI by gender variance in older Chinese community-dwelling adults.

Our research shows a significant correlation between HFHL and CI, and the significance still exists after adjusting for confounding factors. As the most common type of HL in older adults, the incidence of HFHL increases with age, and older people are more likely to have decreased cochlear blood supply and loss of outer hair cells at cochlear basal [26]. HL is one of the most common challenges in older adults over the age of 60 years and a main cause of speech perception [27]. Difficulties in understanding language in older adults result from age-related defects in peripheral and central auditory pathways [13]. Recently, researchers showed that older adults are already using additional cognitive resources for any condition involving speech perception in noise [28]. Whenever the auditory input is degraded either due to HL or due to noise, additional cognitive-control processes are necessary to support speech perception [29]. These studies suggested that there is an inherent connection between HFHL and CI. However, speech audiometry test was not used in this study. Because many studies prefer to use it in those with normal or mild hearing loss, such as an Italian study, they used a speech audiometry test, called Italian version of the Synthetic Sentence Identification-Ipsilateral Competing Message, to assess speech intelligibility central patterns only in PTA ≤ 40 dB HL participants, and merely get a part of the age-related central auditory processing disorder spectrum [30]. But our study tends to focus on older adults whose PTA > 40 dB HL, the correlation between hearing threshold and cognitive function may be more obvious than speech perception ability. Moreover, the MMSE test includes a subtest of language ability that can assess language function. And our results showed it has a significant association with speech-frequency PTA and high-frequency PTA in all participants (*P* = 0.050; *P* = 0.010, respectively), but not in males or females after stratified by gender (all *P* > 0.05). To some extent, our study showed that there is no independent correlation between hearing and language function in males and females. It is worth noting that we are conducting a large cohort study on older adults in Chinese community. Except for those in hospital, nursing homes and other facilities, as well as the bedridden seniors, older adults in the community who could come in for a medical examination were all included in the study. So participants who have communication difficulties or severe cognitive impairment were excluded at recruitment stage, and they were invited to a face-to-face interview with our experienced examiners in quiet environments.

Moreover, several studies have shown that females have a lower hearing threshold and an increased sensitivity compared to males [31, 32]. Although females experience a rapid hearing loss after menopause, the onset of HL is delayed and they have a better hearing function than males of the same age [33, 34]. Our results are similar. This may be related to the protective effect of estrogen on hearing, and the loss of estrogen receptors in the inner ear of males can increase the risk of HL [35]. Our study also shows that females have more sensitive hearing at 4 kHz and 8 kHz but that males have more sensitive hearing at 0.5 kHz and 1 kHz. Similarly, one study showed the same results [36], and another study after adjusting for age using covariance analysis found significant gender differences in pure tone thresholds at 4 kHz and 8 kHz [34]. It is not clear whether these gender differences can solely be attributed to estrogen; however, in measuring hearing, gender as a biological variable has attracted the attention of researchers [15]. It is worth noting that there is a significant correlation between HFHL and CI in females, while similar results can be found merely in males with hypertension (OR: 2.585, 95% Confidence Interval: 1.099–6.082; data not shown). Certainly, there may be gender differences, or other potential mechanisms in the effect of HL on CI may be involved. Moreover, micro-vessel damage may lead to HL, and hypertension is one of the main risk factors of peripheral arterial disease [37]. To some extent, micro-vessel atherosclerosis caused by hypertension may be associated with a reduction in the level of oxygen and the nutrition supply for the inner ear. One study supported the association between hearing and hypertension, particularly at higher frequencies [38]. Another assumed that hypertension may damage not only the inner ear but also the primary auditory cortex [39]. However, a Malaysia study showed that high-frequency PTA (4 kHz and 8 kHz) was not significantly related to cognition [40]. The reason may be due to our subgroup analysis that was performed using gender. Actually, there are few studies on gender differences between HFHL and CI. The results of this cross-sectional study need to be confirmed by additional prospective cohort studies.

Our research also shows a significant correlation between SFHL and CI, and the significance still exists after adjusting for confounding factors. Our results are in line with previous research showing significant associations between greater HL and poorer cognitive function in both cross-sectional and prospective studies [41, 42]. On the contrary, other studies have not found similar results [10, 11]. One major limitation across these previous studies has been how HL was measured and how the variability of audiometric data were analyzed. The strengths of our present study include results from a population-based cohort of older community-dwelling adults and HL adopted by the WHO [43]. The difficulty of speech perception in quiet background is the most prominent feature of SFHL in older adults. A previous study demonstrated that older adults with HL showed reduced recruitment of the articulatory motor cortex during listening to speech at 0.5 to 4 kHz versus whom with normal hearing [40]. The older adults with HL were also damaged in speech perception in noise background. The current findings suggest that auditory input from the cochlear to the auditory system in older adults are reduced, which leads to a reduced recruitment of the articulatory motor system in speech processing and supports the auditory-motor decline hypothesis [44]. Communication disorders caused by HL can result in social isolation and loneliness in older adults, and many epidemiologic and neuroanatomic studies have supported correlations between loneliness and CI. The effect of HL on cognitive load is suggested by studies indicating that under conditions of auditory perception is difficult, and more cognitive resources are devoted to auditory perceptual processing, thus damaging other cognitive processes such as working memory [45]. Neuroimaging studies had already indicated that older adults have a compensatory recruitment of regions in their prefrontal and temporoparietal cortex, which to maintain auditory speech processing [27, 46]. And this pattern of neural compensation may explain the general preservation of language comprehension, which is seen even in the advanced dementia population [47].

In our study, male’s high-frequency PTA worsened with each older age group (40.66 ± 11.72 vs 43.59 ± 12.58 vs 49.40 ± 14.50; *P* < 0.001; data not shown); their speech-frequency PTA also worsened with each older age group. In females, we found similar results. However, those 75 years and older, SFHL and HFHL were not significantly associated with CI in males and females. Because our study population was older and the hearing function of old adults is generally weakened not continuously getting worse in aging which described as the ceiling effect [48]. Our older old participants were more prone to a higher prevalence of HL. Exactly, the older old, especially with HL, whose hearing function may not significantly with CI anymore for high prevalence of HL. This is consistent with the results of a previous study [11]. As for gender differences of hearing function, they were almost minimal in the older old [48]. Their cognitive function is inevitably affected by many chronic diseases especially in older old; we cannot rule out other potential confounders contributing to CI in this cross-sectional study. Interestingly, we found that the OR value in females with SFHL or HFHL seemed to have a trend of decreasing with increasing age, and this trend was more obvious in pre-elderly females (< 75 years), but we did not observe any similar results in pre-elderly males. This is different from a previous study [49]. The reason may be due to different hearing loss definition and assessment tools. Besides, we have another two possible explanations: one may be due to the heterogeneity of population: the association between HL and CI was stronger in our pre-elderly; another could be for the relatively small sample size after stratification by age. We plan to increase the sample size in the future, which may produce new results.

We found that impairment on the MMSE overall is independently and significantly negatively related to hearing sensitivity after adjusting for confounding variables. Hearing sensitivity is also negatively related to hearing status. Similarly, one report by Lin et al. demonstrated that those with HL performed worse in MMSE scores than those with normal hearing [50]. In the “Registration” sections of MMSE, high- and speech-hearing sensitivity has a significantly negative correlation with registration scores in males and females. The causes of cognitive decline in adults with HL could be explained by the information degradation hypothesis, and HL can be interpreted as that to put an increased burden on cognitive processing on account of the effort required to decode the degraded sensory input [51]. Other studies have approved that performance in cognitive tests might be affected by the quality of auditory‐presented sensory input such as memory [52]. Moreover, time, recall, and attention/calculation sections of MMSE have a significantly negative correlation with speech-hearing sensitivity in males after adjusting for confounding variables. This may be due to further advancement in the HL pathophysiological process, and speech-frequency hearing sensitivity was related to broader cognitive domains than high-frequency hearing sensitivity. Similarly, a meta-analysis reported that HL was associated with cognitive decline involving multiple domains, including working memory and visuospatial ability [9]. It is noteworthy that high-/speech-hearing sensitivity was not significantly correlated with language scores in males and females. A cross-sectional study also analyzed the possible relationship between hearing sensitivity and cognition on language tests, which shown there are no significant relationship between hearing sensitivity and speech function, regardless of CI [53]. These results suggested that cognitive function, rather than simply auditory problems, is attributed to the impaired speech function in older adults.

## Limitations

This study had several limitations. First, as this was a cross-sectional study design, although correlations among SFHL, HFHL, and CI were found in older adults, the causation is still unknown. Second, all of our subjects are from four communities in Chongming District, Shanghai, and we required them to arrive at the prescribed place of physical examination by themselves and provide their informed consent; hence, subjects with severe CI were not included in our study. In the future, we will improve our research design and more accurately ascertain the association between CI and HL at different frequencies among older community dwellers in China.

## Conclusion

Our study suggests that SFHL and HFHL are associated with CI, but males and females demonstrate different results, suggesting that different mechanisms are involved in the etiology of HL. Moreover, we found that hearing sensitivity is negatively associated with cognitive domains, and that registration is most significant. The associations between hearing frequencies and cognitive health may vary according to gender. Gender-specific strategies in healthcare policies are needed. In view of our limited ways to assess cognitive function, future research should focus on a more comprehensive approach to assessing cognition and implementing a longitudinal study in order to explore the causal relationships between HL and CI.

## Data Availability

The datasets generated during and/or analyzed during the current study are available from the corresponding author on reasonable request.

## References

[1] GBD (2016). Dementia Collaborators (2019) Global, regional, and national burden of Alzheimer's disease and other dementias, 1990–2016: a systematic analysis for the Global Burden of Disease Study 2016. Lancet Neurol.

[2] Wang J, Xiao LD, Wang K (2020). Gender Differences in Cognitive Impairment among Rural Elderly in China. Int J Environ Res Public Health.

[3] Jia J, Wei C, Chen S (2018). The cost of Alzheimer's disease in China and re-estimation of costs worldwide. Alzheimer's Dementia.

[4] Norton S, Matthews FE, Barnes DE (2014). Potential for primary prevention of Alzheimer's disease: an analysis of population-based data. Lancet Neurol.

[5] Baumgart M, Snyder HM, Carrillo MC (2015). Summary of the evidence on modifiable risk factors for cognitive decline and dementia: A population-based perspective. Alzheimer's Dementia.

[6] Gallacher J, Ilubaera V, Ben-Shlomo Y (2012). Auditory threshold, phonologic demand, and incident dementia. Neurology.

[7] Lin FR, Albert M (2014). Hearing loss and dementia - who is listening?. Aging Ment Health.

[8] Panza F, Lozupone M, Sardone R (2018). Sensorial frailty: age-related hearing loss and the risk of cognitive impairment and dementia in later life. Therapeutic Adv Chronic Dis.

[9] Loughrey DG, Kelly ME, Kelley GA (2018). Association of age-related hearing loss with cognitive function, cognitive impairment, and dementia: a systematic review and meta-analysis. JAMA Otolaryngol Head Neck Surg.

[10] Lin MY, Gutierrez PR, Stone KL (2004). Vision impairment and combined vision and hearing impairment predict cognitive and functional decline in older women. J Am Geriatr Soc.

[11] Gussekloo J, de Craen AJ, Oduber C (2005). Sensory impairment and cognitive functioning in oldest-old subjects: the Leiden 85+ Study. Am J Geriatric Psychiatry.

[12] Tun PA, Williams VA, Small BJ (2012). The effects of aging on auditory processing and cognition. Am J Audiol.

[13] Gates GA, Mills JH (2005). Presbycusis. Lancet (London, England).

[14] Kim B, Oh SH (2013). Age-related changes in cognition and speech perception. Korean J Audiol.

[15] Nolan LS (2020). Age-related hearing loss: Why we need to think about sex as a biological variable. J Neurosci Res.

[16] Delhez A, Lefebvre P, Péqueux C (2020). Auditory function and dysfunction: estrogen makes a difference. CMLS.

[17] Lee FS, Matthews LJ, Dubno JR (2005). Longitudinal study of pure-tone thresholds in older persons. Ear Hear.

[18] Kiely KM, Gopinath B, Mitchell P (2012). Cognitive, health, and sociodemographic predictors of longitudinal decline in hearing acuity among older adults. The journals of gerontology. Series A Biolog Sci Med Sci.

[19] WHO Programme for the Prevention of Blindness and Deafness: WHO ear and hearing disorders survey. https://apps.who.int/iris/handle/10665/67892. Accessed 17 June 2012.

[20] Zhang MY, Katzman R, Salmon D (1990). The prevalence of dementia and Alzheimer's disease in Shanghai, China: impact of age, gender, and education. Ann Neurol.

[21] Jiang CQ, Xu L, Lam TH (2009). Zhonghua liu xing bing xue za zhi. Zhonghua liuxingbingxue zazhi.

[22] Katz S, Ford AB, Moskowitz RW (1963). Studies of illness in the aged. The index of ADL: a standardized measure of biological and psychosocial function. JAMA.

[23] Lawton MP, Brody EM (1969). Assessment of older people: self-maintaining and instrumental activities of daily living. Gerontologist.

[24] Vivanco-Hidalgo RM, Elosua R, Gómez González A (2017). People with epilepsy receive more statins than the general population but have no higher cardiovascular risk: results from a cross-sectional study. Eur J Neurol.

[25] Brinkley TE, Leng X, Miller ME (2009). Chronic inflammation is associated with low physical function in older adults across multiple comorbidities. The journals of gerontology. Series A Biolog Sci Med Sci.

[26] Peelle JE, Wingfield A (2016). The neural consequences of age-related hearing loss. Trends Neurosci.

[27] Peelle JE, Troiani V, Grossman M (2011). Hearing loss in older adults affects neural systems supporting speech comprehension. J Neurosci.

[28] Wingfield A, Tun P, McCoy S (2005). Hearing loss in older adulthood: what it is and how it interacts with cognitive performance. Curr Dir Psychol Sci.

[29] Houtgast T, Festen JM (2008). On the auditory and cognitive functions that may explain an individual's elevation of the speech reception threshold in noise. Int J Audiol.

[30] Sardone R, Battista P, Donghia R et al (2020) Age-Related central auditory processing disorder, MCI, and dementia in an older population of Southern Italy. Otolaryngol Head Neck Surg 163:348–355. 10.1177/0194599820913635 10.1177/0194599820913635 32312167

[31] Hoffman HJ, Dobie RA, Losonczy KG (2017). Declining prevalence of hearing loss in US adults aged 20 to 69 years. JAMA Otolaryngol Head Neck Surg.

[32] Snihur AW, Hampson E (2011). Sex and ear differences in spontaneous and click-evoked otoacoustic emissions in young adults. Brain Cogn.

[33] Jönsson R, Rosenhall U, Gause-Nilsson I (1998). Auditory function in 70- and 75-year-olds of four age cohorts. A cross-sectional and time-lag study of presbyacusis. Scand Audiol.

[34] Kim S, Lim EJ, Kim HS (2010). Sex differences in a cross sectional study of age-related hearing loss in Korean. Clinical and experimental otorhinolaryngology.

[35] Hultcrantz M, Simonoska R, Stenberg AE (2006). Estrogen and hearing: a summary of recent investigations. Acta Otolaryngol.

[36] Pearson JD, Morrell CH, Gordon-Salant S (1995). Gender differences in a longitudinal study of age-associated hearing loss. J Acoust Soc Am.

[37] Matsunaga M, Yatsuya H, Iso H (2017). Similarities and differences between coronary heart disease and stroke in the associations with cardiovascular risk factors: The Japan Collaborative Cohort Study. Atherosclerosis.

[38] Agarwal S, Mishra A, Jagade M (2013). Effects of hypertension on hearing. Indian J Otolaryngol Head Neck Surg.

[39] Umesawa M, Sairenchi T, Haruyama Y (2019). Association between hypertension and hearing impairment in health check-ups among Japanese workers: a cross-sectional study. BMJ Open.

[40] Mukari S, Ishak WS, Maamor N (2017). A preliminary study investigating the association between hearing acuity and a screening cognitive tool. Ann Otol Rhinol Laryngol.

[41] Valentijn SA, van Boxtel MP, van Hooren SA (2005). Change in sensory functioning predicts change in cognitive functioning: results from a 6-year follow-up in the maastricht aging study. J Am Geriatr Soc.

[42] Peters CA, Potter JF, Scholer SG (1988). Hearing impairment as a predictor of cognitive decline in dementia. J Am Geriatr Soc.

[43] WHO: Prevention of Deafness and Hearing Impaired Grades of Hearing Impairment. https://www.who.int/pbd/deafness/hearing_impairment_grades/en/. Accessed 25 November 2013.

[44] Panouillères M, Möttönen R (2018). Decline of auditory-motor speech processing in older adults with hearing loss. Neurobiol Aging.

[45] Füllgrabe C (2020). On the possible overestimation of cognitive decline: the impact of age-related hearing loss on cognitive-test performance. Front Neurosci.

[46] Wingfield A, Grossman M (2006). Language and the aging brain: patterns of neural compensation revealed by functional brain imaging. J Neurophysiol.

[47] Rousseaux M, Sève A, Vallet M (2010). An analysis of communication in conversation in patients with dementia. Neuropsychologia.

[48] Wattamwar K, Qian ZJ, Otter J (2017). Increases in the rate of age-related hearing loss in the older old. JAMA Otolaryngol Head Neck Surg.

[49] Osler M, Christensen GT, Mortensen EL (2019). Hearing loss, cognitive ability, and dementia in men age 19–78 years. Eur J Epidemiol.

[50] Lin FR, Ferrucci L, Metter EJ (2011). Hearing loss and cognition in the Baltimore Longitudinal Study of Aging. Neuropsychology.

[51] Wayne RV, Johnsrude IS (2015). A review of causal mechanisms underlying the link between age-related hearing loss and cognitive decline. Ageing Res Rev.

[52] McCoy SL, Tun PA, Cox LC (2005). Hearing loss and perceptual effort: downstream effects on older adults' memory for speech. The Quarterly journal of experimental psychology. A Hum Experim Psychol.

[53] Lodeiro-Fernández L, Lorenzo-López L, Maseda A (2015). The impact of hearing loss on language performance in older adults with different stages of cognitive function. Clin Interv Aging.

